# A call for preregistration of in vitro research: Pregistration as a tool to improve reproducibility and transparency

**DOI:** 10.1038/s44319-026-00764-x

**Published:** 2026-04-17

**Authors:** Céline Heinl, Nuno H Franco, Bettina Bert, Katherina Siewert, Dania Movia, Edlira Aruçi, Carina Ladeira, Ahmed Y Sanin, Winfried Neuhaus, Ulf D Kahlert, Manosij Ghosh, Aurélie Thomas, Athanassia Sotiropoulos

**Affiliations:** 1 German Centre for the Protection of Laboratory Animals (Bf3R) at the German Federal Institute for Risk Assessment (BfR), Berlin, Germany; 2 i3S - Instituto de Investigação e Inovação em Saúde, Universidade do Porto, Porto, Portugal; 3 Department of Chemical and Product Safety, German Federal Institute for Risk Assessment (BfR), Berlin, Germany; 4 Department of Biology and Kathleen Lonsdale Institute for Human Health Research, Maynooth University, Callan Building, Maynooth, Ireland; 5 Trinity St. James’s Cancer Institute, Radiobiology and Molecular Oncology Research Group, Applied Radiation Therapy Trinity, Discipline of Radiation Therapy, Trinity College Dublin, Dublin, Ireland; 6 Department of Biotechnologies, Chemistry and Pharmacy, University of Siena, Siena, Italy; 7 Escola Superior de Saúde de Lisboa, Polytechnic University of Lisbon, Lisbon, Portugal; 8 Comprehensive Health Research Centre (CHRC), National School of Public Health, NOVA University of Lisbon, Lisbon, Portugal; 9 Medical Faculty and University Medical Center Magdeburg, Clinic for General-, Visceral-, Vascular-, and Transplantation Surgery, Magdeburg, Germany; 10 Competence Unit Molecular Diagnostics, Center for Health and Bioresources, AIT-Austrian Institute of Technology GmbH, Vienna, Austria; 11 Faculty of Medicine and Dentistry, Danube Private University, Krems, Austria; 12 Research Campus STIMULATE, Otto-Von-Guericke University Magdeburg, Magdeburg, Germany; 13 Department of Public Health and Primary Care, Centre for Environment and Health, Katholieke Universiteit Leuven, Leuven, Belgium; 14 Animal Sciences & Technology, Clinical Pharmacology and Safety Sciences, R&D, AstraZeneca, Cambridge, UK; 15 Inserm, GIS FC3R, 94700 Maisons-Alfort, France

**Keywords:** Economics, Law & Politics, Science Policy & Publishing

## Abstract

This article highlights the benefits of preregistration in improving the reliability and transparency of in vitro studies. By preregistering their studies, researchers can make their findings more reproducible, paving the way for medical progress, and accelerating the replacement of animal experiments.

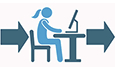

Biomedical research, as any other research field, is vulnerable to problems in its planning, execution, documentation, analysis, interpretation, and reporting. These issues can impede the reproducibility of research findings and hinder translation into clinical practice.

In addition, there is growing societal pressure to hasten the shift to animal-free research methods (Pound and Ritskes-Hoitinga, 2018). To alleviate the ethical dilemma of using animals in research, the 3Rs principle (replace, reduce, refine) calls for reducing or replacing such experiments wherever possible. Nevertheless, scientific and biomedical research, as well as safeguarding human and environmental health, continue to depend on animal experiments. To apply the 3Rs principles without compromising scientific progress and safety standards, it is imperative to ensure that in vitro methods to replace animal experiments are robust and reliable.

“To apply the 3Rs principles without compromising scientific progress and safety standards, it is imperative to ensure that in vitro methods to replace animal experiments are robust and reliable.”

Moreover, research in general, and in vitro research in particular, is often funded by public funds. This places a responsibility on scientists to make all collected data publicly available, including “negative” or “inconclusive” data.

Preregistration aims to address these problems and improve the quality of published research. Registering a study plan before conducting experiments— including hypotheses, data-collection procedures, and analysis plan reduces the risk of publication bias and questionable research practices and makes the research process more transparent. At present, however, preregistration is only mandatory for clinical trials, though it has increasingly been used in other research fields, notably psychology and animal research.

## Preregistration to improve robustness and reliability

We suggest that preregistration has sufficient potential and advantages to be applied to in vitro research. With the emergence of innovative methodologies, such as organoids and organs-on-chips, which can increasingly mimic the functions of human organs to replace animal experiments, it is imperative to ensure that results are robust and reliable, thereby accelerating their integration into fundamental and translational research.

Currently, however, there is no dedicated registry for preregistering in vitro research. We propose developing a preregistration framework adapted to in vitro studies. We recommend establishing a task force composed of researchers, funding bodies, journals, and organizations dedicated to promoting preregistration and setting up a pragmatic preregistration process for any kind of research relying on in vitro methods. This process should cater to researchers’ needs and ensure high acceptance within the research community.

Preregistration is an effective tool for addressing flaws in research quality, publication bias, and avoiding unnecessary repetition of experiments. It was initiated in clinical trials and later adopted by animal research with the objective of enhancing the robustness of research findings by registering a time-stamped study plan prior to data collection with clear rationales for hypotheses and analytical approaches (Nosek et al, 2018; Lakens et al, 2024; Kaplan and Irvin, 2015).

Moreover, preregistration has the potential to address publication bias by encouraging the dissemination of all results, including those that do not support the hypothesis. In accordance with the principle that in vitro research should be subjected to the same quality standards as other methodologies, we suggest that it should also be preregistered. We argue that time-stamped declaration of study intent, specification of primary outcomes, and transparent tracking of deviations can improve biomedical research broadly, whether conducted in vivo or in vitro. We therefore suggest the inclusion of an in vitro preregistration framework into existing registries for animal research to ease implementation. A differentiation between confirmatory and exploratory studies could facilitate practicability and improve acceptance in the research community.

## Current quality issues in in vitro research

The reproducibility crisis is not exclusive to preclinical biomedical research but a more general phenomenon that afflicts numerous disciplines (Baker, 2016). One of the major causes of poor reproducibility is a lack of sufficient experimental detail in reporting (Sander et al, 2022). An open investigation of the reproducibility of preclinical cancer studies, encompassing both in vivo and in vitro experiments, revealed that none of the 193 methods could be replicated based solely on the original manuscripts; each required some degree of clarification from the original authors (Errington et al, 2021a). This issue has been addressed for regulatory use in human safety assessment by the Guidance Document on Good in vitro Method Practices (GIVIMP), the Good Cell Culture Practices (GCCP 2.0), which incorporate guidelines on the use of microphysiological systems, and, more recently, the DRIVER (Designing and Reporting In Vitro Experiments Responsibly) recommendations by the UK National Centre for the Replacement, Refinement & Reduction of Animals in Research (NC3Rs) that provide guidance for various types of in vitro research.

Nevertheless, better reporting of experiment detail alone is inadequate to redress deficiencies in study design. The act of reporting typically starts when the research process ends and therefore cannot solve problems arising before. The cancer reproducibility project has in fact identified additional quality issues: specifically, only 54% of the replicated in vitro experiments exhibited a similar effect as the original study. Moreover, the effect sizes of the replicated results were overall smaller than those reported in the original publication (Errington et al, 2021b). These findings indicate researchers’ unconscious bias towards the publication of “positive results”, which is favored particularly by high-impact journals. Although this selective reporting of positive findings is not driven by malicious intent, it undermines the foundations of empirical biomedical research (Sena et al, 2010). The non-publication of negative results not only distorts the evidence regarding a given effect or phenomenon but also leads to a waste of time and financial resources, as multiple laboratories may unknowingly try the same unsuccessful approach.

This bias is further reinforced by questionable, although not necessarily intentionally fraudulent, research practices such as p-hacking and HARKing. In brief, p-hacking occurs when researchers exploit analytical flexibility or degrees of freedom to obtain a desired small p-value, while HARKing occurs when researchers scan large datasets without a predefined hypothesis to find a significant effect and claim the one found was what they were looking for, or when they change the previous hypothesis to a new one based on the results obtained, claiming it was the original one being tested. Although these issues are primarily discussed in the context of animal research, recent studies suggest that in vitro research is similarly affected (Errington et al, 2021b).

## Benefits of preregistration

**Figure 1:**
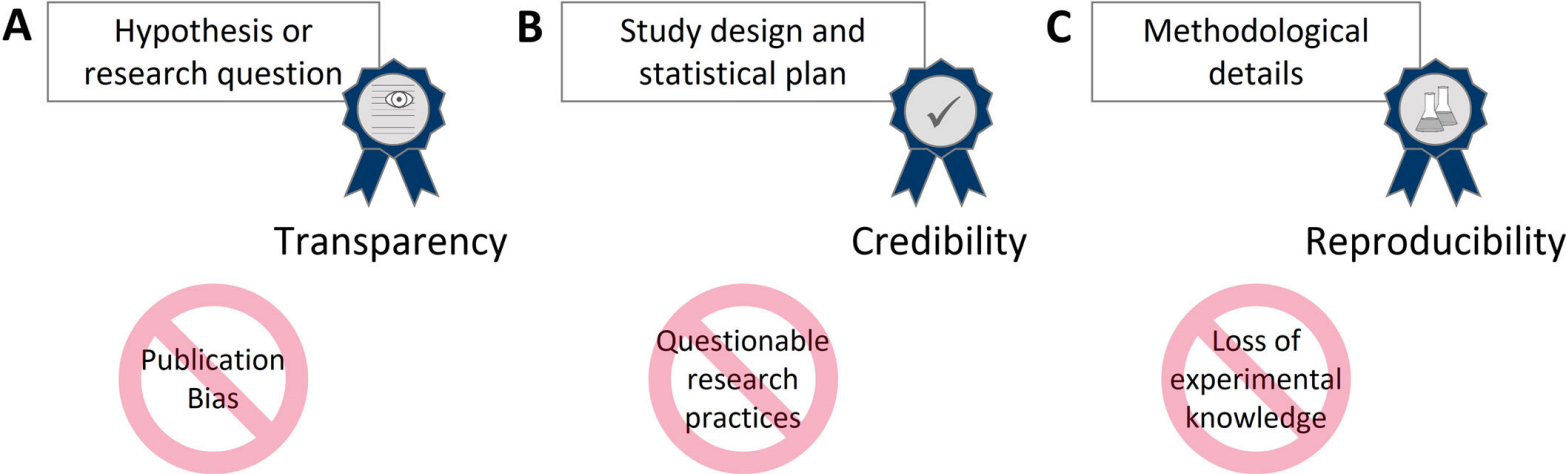
Preregistration can improve research regarding. (**A**) Transparency—depositing hypotheses or research questions in a public registry ensures openness and helps prevent selective reporting and HARKing. It moreover can reduce publication bias, by leaving a public trace of each preregistered study, regardless of outcome. (**B**) Credibility—preregistering the study design and statistical plan reduces the risk of questionable research practices, such as p-hacking. (**C**) Reproducibility—documenting methodological details (according to harmonized standards) ensures studies can be replicated, and that failed methods are recorded, avoiding loss of experimental knowledge.

Registration of a time-stamped study plan prior to starting in vitro experiments could address the quality issues described above (Sander et al, 2022). The benefits of preregistration (Fig. 1) include:

### Reducing publication bias

By preregistering a hypothesis or research question, preregistered studies leave a public trace and make potential publication bias visible.

### Careful reflection on experimental design and prevention of questionable research practices such as p-hacking and HARKing

Templates include fields on the hypotheses to be tested, the predictions made, the fixed and random factors at play, the groups compared, the controls, the sample size, and the method for blinding and randomization. In addition, the preregistration of an analysis plan prior to data collection can prevent analytical flexibility and data-driven decision-making—for instance, by defining exclusion criteria based on the results, selecting another group as control, changing the statistical test, adding more replicates, transforming the data, and so on—hereby reducing the risk of biased or spurious findings (Kaplan and Irvin, 2015; van den Akker et al, 2024).

### Preserve knowledge on methods used and promote comprehensible reporting

Preregistration templates covering the most crucial details of the methodology help researchers with good reporting during the final publication.

Preregistration was first introduced in clinical research in a top-down approach to increase the transparency and reliability of clinical trials (Al-Durra et al, 2020). Governmental institutions have established dedicated platforms for clinical-trial registration, with ClinicalTrials.gov, developed by the US Food and Drug Administration, as the most prominent example. In certain regions, including the USA, registering eligible clinical trials on such platforms is mandated by regulatory authorities. Alongside these governmental requirements, journals, funding agencies, and international guidelines have strongly encouraged trial registration, accelerating its widespread adoption. Now, more than twenty years since trial preregistration was introduced, it has become a well-established standard in clinical research, the importance of which is widely acknowledged. Registration has contributed to greater transparency in reporting outcomes and may shift research findings toward more null results, providing a more accurate representation of clinical effects (Kaplan and Irvin, 2015).

“… more than twenty years since trial preregistration was introduced, it has become a well-established standard in clinical research, the importance of which is widely acknowledged.”

In contrast, preregistration in the social sciences was introduced through a bottom-up approach when psychology was hit hard by the reproducibility crisis. Scientists recognized the need for change, leading to the introduction of community-based initiatives and new preregistration platforms, such as the *Open Science Framework* registry (https://osf.io/) or *As predicted* (https://aspredicted.org/). Publishers supported this paradigm shift by introducing Registered Reports, a format now offered by more than 300 journals (Chambers, 2013). This new publication format involves peer-review of protocols of designed studies before data are collected, evaluating the idea and the rigor of the methodology rather than the study results. Peer-reviewed protocols can be accepted *in principle*, which means that the study will be published if the research is carried out in accordance with the protocol.

In the social sciences, preregistration has been the subject of intense debate regarding its benefits and limitations. Critics raised concerns that it might put additional burdens on authors and reviewers. Furthermore, labeling studies as preregistered could give the impression that these papers are particularly trustworthy, when this may not be warranted (Krishna, 2021). Advocates of preregistration point to the huge increase in voluntary preregistrations in recent years and clarified that exploratory research is not slowed down, but rather transparently labeled as such. Preregistration is not in itself a seal of quality, but it does enable readers to make an informed judgment about the validity of a study. Psychology is a good example of how transparent discussions about what preregistration can and cannot achieve help to broaden its acceptance and implementation.

“Psychology is a good example of how transparent discussions about what preregistration can and cannot achieve help to broaden its acceptance and implementation.”

In the biomedical field, preregistration has only recently been introduced, specifically for animal research, after data highlighted the poor translation of preclinical animal results into clinical outcomes, and reports of unpublished negative results. In response, two preregistration platforms specifically developed for animal research were launched: *preclinicaltrials.eu* and *animalstudyregistry.org* (Arrowsmith and Miller, 2013; Wieschowski et al, 2019; Heinl et al, 2020). Although preregistration is still marginal in animal research with 536 protocols registered across both registries as of January 19, 2026, we have seen a steady uptake over the last years. A recent study found that preregistration significantly enhances the quality of animal research by improving internal validity and increasing consistency in reporting (Menon et al, 2026).

## Current state of preregistration of in vitro research

General preregistration platforms such as the *Open Science Framework* registry (https://osf.io/) or *As predicted* (https://aspredicted.org/) were initially developed for social science and psychological research, and may not fully address the specific needs of the life sciences. *Animalstudyregistry.org*, originally designed for animal research, allows for the preregistration of in vitro research when it is complementary to an animal study. The European Food Safety Agency (EFSA) has also developed a platform on which applicants seeking approval for new food-related products must register studies investigating product safety. This preregistration step is independent of the methods used and could therefore include in vitro studies. However, it is limited to studies conducted specifically for regulatory approval. There is currently no open preregistration platform for studies exclusively dedicated to in vitro research.

Wherever preregistration was introduced, it has triggered lively discussions within the relevant communities about what kind of research should be preregistered. For in vitro research, the most practical distinction can be made between exploratory studies, which are often designed in the absence of a clear predefined hypothesis, and confirmatory studies, which aim to test a specific preconceived hypothesis (Dirnagl, 2020). We suggest that both should be preregistered, using appropriate templates.

Preregistration of in vitro confirmatory research is very straightforward because it is based on previous results. Key methodological details, such as expected effect sizes and experimental parameters of an already established method, can be easily defined a priori.

**Figure 2:**
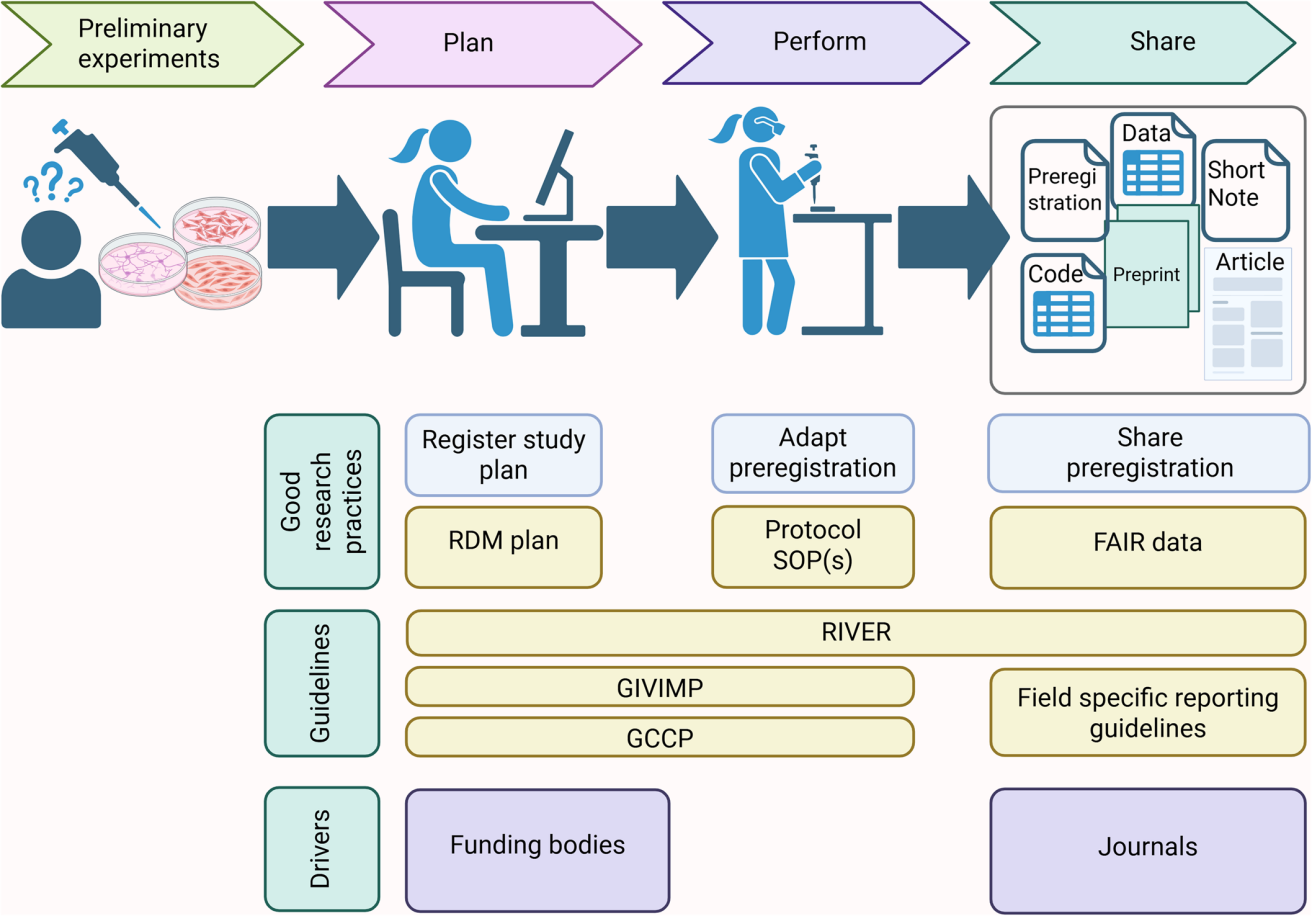
Integrating preregistration into the research process. Preregistration aligns with RIVER, GIVIMP, and GCCP and should occur after pilot experiments (exploratory) or before data collection (confirmatory). Preregistration may remain hidden during an embargo period to prevent *scooping*. Entries can be adapted anytime, and unforeseen changes will become visible. Once the study is completed, the embargo should be lifted, and all information can be shared and linked to the preregistration. This includes more publication formats than classical journal articles such as preprints, datasets or short notes, specifically conceived for negative or inconclusive data (https://www.fc3r.com/en/FC3R-short-notes.php). As preregistration is implemented at the beginning of the research process, funders could be the main drivers for the initiation of preregistration for in vitro studies.

Preregistration of exploratory research is more controversial and methodologically challenging (Dirnagl, 2020). As exploratory studies are designed to generate rather than test hypotheses, they typically allow greater flexibility in design and analysis, which can unintentionally increase the risk of irreproducible findings if not transparently documented. Preregistration should be adapted to accommodate exploratory research by using shorter, more flexible registration formats that suit its iterative and discovery-driven nature, while still maintaining transparency and accountability (Fig. 2).

“Preregistration should be adapted to accommodate exploratory research by using shorter, more flexible registration formats that suit its iterative and discovery-driven nature…”

To ensure that data can meaningfully inform future advances in human health, preclinical studies performed for regulatory purposes, such as those supporting applications for first-in-human trials, are subject to rigorous oversight, including formal requirements like Good Laboratory Practice (GLP). The implementation of preregistration in this context seems natural and could further reduce the risks for human subjects. In contrast, most exploratory in vitro research does not fall under these regulatory frameworks, although such studies may still be subject to institutional guidelines, ethical review, and transparency requirements. It is essential to note that exploratory research is not synonymous with low-quality research but requires more flexibility to accommodate new discoveries and iterative refinement. Large-scale sequence generation, screening approaches, observational in vitro studies, and systems-biology projects are often explicitly hypothesis-generating, where the primary goal is to identify novel patterns, relationships, or candidates for further investigation. Nevertheless, given its scale and reliance on public funding, this research should be held to high standards. In addition, many in vitro studies lead to animal testing downstream, which should be based on robust data to avoid animal use without a significant chance of a valuable outcome. Enabling the preregistration of exploratory in vitro research could therefore contribute to the sustainable development of animal-free methods by adding robustness and credibility to published results.

For these types of studies, preregistration should not impose artificial constraints. Instead, a simple format could document the study rationale, and the planned methodological approach and analysis of data. Explicitly labeling studies as exploratory could provide two benefits. First, it increases transparency regarding analytic flexibility by documenting planned analysis, including bioinformatic preprocessing and discouraging selective reporting. Second, it can prevent exploratory findings from being reframed as confirmatory evidence after conclusion, thereby giving the results a higher validity. Strengthening the transparency and robustness of results from exploratory research can increase the likelihood of subsequent replication, accelerate the implementation of in vitro methodologies, and support translational efforts.

For industry, preregistration may have additional appeal: small biotech firms, in particular, often need to establish credibility of their research and results to attract investors and facilitate product sales. By preregistering trials, companies can strengthen confidence in their findings and foster greater trust among potential partners and investors. Pharmaceutical companies also stand to benefit from robust and transparent trial outcomes. Nonetheless, to be widely adopted, preregistration must accommodate industry realities, which include protecting IP while avoiding premature disclosure that could erode competitive advantage via calibrated embargoes and selective, time‑stamped disclosures aligned with patent and market timelines.

In summary, the greater the expected impact of a study, the more important preregistration becomes. This is particularly important if the study is to serve as a basis for research involving human subjects. Nevertheless, preregistration is also valuable for other research with less immediate impact, such as exploratory in vitro studies.

“… the greater the expected impact of a study, the more important preregistration becomes.”

## How could preregistration be adapted to in vitro research?

In vitro studies represent a diverse range of methodological approaches widely used in preclinical biomedical research. Preregistration does therefore not necessarily require the creation of new registries. In fact, we should not artificially widen the gap between complementary methodologies. Therefore, the implementation of in vitro preregistration in existing animal registries would be desirable, as using existing infrastructure and the experience of those who manage it would help ensure the implementation of common quality standards established by different registries (Heinl et al, 2022).

The essential features of preregistration are well defined by established registries. To protect research concepts in a competitive landscape, an embargo period allows the main content of the protocol to stay hidden temporarily. Preregistration platforms also record time-stamped updates, enabling researchers to document the rationale for study amendments or the addition of new experiments. Linking preregistrations to final outcomes is crucial and can be achieved by referencing publications, connecting to data repositories, or directly uploading results. All these features should be incorporated into preregistration tools designed for in vitro studies.

Expanding preregistration to in vitro research will require the development or adaptation of templates that reflect its unique requirements, in particular the methods section (Fig. 1). These templates should be specific enough to ensure rigorous planning and capturing all important details, but flexible enough to accommodate diverse methodologies without imposing unnecessary constraints. Well-structured preregistration forms will also help to build search tools that can make preregistrations a valuable source of information for the research community. Researchers could also be supported by establishing a new preregistration checklist when planning their experiments, similar to the PREPARE concept in animal research (https://norecopa.no/PREPARE). While confirmatory studies can and should preregister more details to increase reliability, exploratory studies should have the possibility of a shorter version covering only the essential predictable items.

“While confirmatory studies can and should preregister more details to increase reliability, exploratory studies should have the possibility of a shorter version covering only the essential predictable items.”

A possible approach is to have a universal preregistration template accompanied by specific examples of different types of in vitro models, methodologies, and the context of use. This would avoid the need for multiple templates and allow flexibility, while ensuring broad applicability and guidance along with some level of harmonization among submissions. Another advantage of having examples rather than multiple templates would be that new examples could be added by the research community without affecting the overall structure of the template.

In our proposed framework for a universal preregistration form, researchers should be able to preregister multiple methods for any given study, regardless of whether these are animal-based or in vitro methods, as these approaches are often complementary within a project. Such a framework should allow proportional preregistration: detailed hypotheses and analysis plans for confirmatory studies, and structured documentation of design choices and decision criteria for exploratory work. Existing reporting guidelines, such as the DRIVER recommendations for in vitro research, can serve as a valuable basis for defining key fields to be included in the preregistration form (https://nc3rs.org.uk/our-portfolio/driver-recommendations).

Alternative options for preregistering in vitro studies beyond traditional registries are also conceivable. For example, the NC3Rs *Experimental Design Assistant* helps scientists to plan animal studies and obtain a time-stamped study plan (https://eda.nc3rs.org.uk/). Adapting this platform to include in vitro research could broaden its appeal and promote non-animal methods to animal users. Platforms such as *protocols.io*, designed specifically for sharing methodological protocols, are very user-friendly and could be used for preregistration of methods (https://www.protocols.io/). Protocols stored in *protocols.io* are time-stamped and can remain private until researchers decide to share them publicly. However, we should keep in mind that the more different formats are used for preregistration, the more complex the usability and searchability of preregistrations will be.

## Barriers and strategies for implementing preregistration of in vitro research

The successful implementation of preregistration requires broad acceptance by the respective research community. For animal preregistration, which was introduced seven years ago, adoption is still low. While there are several reasons for this, early involvement of a representative group of diverse animal researchers in the development of the preregistration tool might have helped to increase its acceptance and use.

To address this issue, an initiative by qualitative researchers used the Delphi method, an iterative process of expert knowledge elicitation through questionnaires with feedback, to design a tailored template (Haven et al, 2020). A similar approach could be applied to in vitro research, engaging researchers, journals, funders, institutions, and platform providers to identify key information to maximize reproducibility while minimizing requirements, ultimately distilling them down to the absolute essentials. This task force could help develop the preregistration template and define key features, such as the length of the embargo period. Such a community-driven approach would not only meet the needs of researchers but also increase support for preregistration among industry advocates.

**Figure 3:**
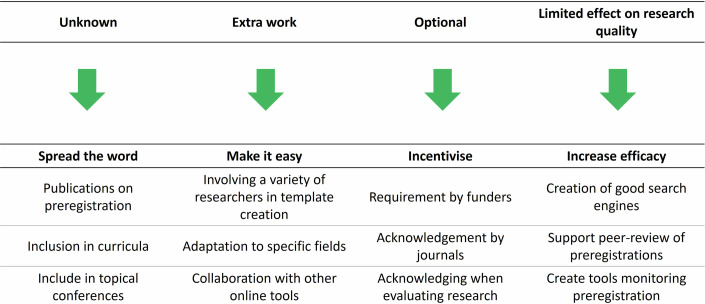
Challenges and strategies for implementing preregistration in in vitro research. Early lessons from other disciplines highlight the need for strong communication, lean templates with data reuse to reduce workload, and incentives from funders, journals, and institutions to achieve critical uptake. Continuous evaluation, cross-registry search, reviewer aids, and automated tracking of preregistered studies can maximize benefits for research quality.

Once a preregistration format has been established, it will be critical to raise awareness within the research community in order to achieve its uptake (Fig. 3). In addition to using traditional channels, such as publications, an effective communication strategy could be to target early-career researchers. They are known to be more receptive to open-science practices and could play a crucial role in changing the way in vitro science is conducted (Kent et al, 2022). Thus, preregistration could therefore be incorporated into courses on research integrity for graduate students along with topics such as data management or scholarly publishing. Indeed, a significant obstacle to preregistration is the lack of adequate training in robust statistical and methodological practices (Nosek et al, 2018).

“… preregistration could therefore be incorporated into courses on research integrity for graduate students along with topics such as data management or scholarly publishing.”

We recognize that the implementation of preregistration tools in any research process always comes with an additional workload—this is also the main concern of researchers working with animals (Priboi et al, 2025). Shorter templates adapted for exploratory studies could reduce the burden in in vitro as well as in vivo research. In addition, connecting different open-science tools to share information could be a way to reduce the effort required. Import options from grant application forms, electronic lab notebooks, or method protocol platforms, as well as export options to journal reporting checklists, could help to avoid the need to enter the same information multiple times in different places. Dedicated Large Language Models and automation platforms could further facilitate this process.

However, some additional effort will be required for the successful implementation of in vitro preregistration. In clinical research, this came primarily through mandatory requirements by funders, legislation, and scientific journals. Similar approaches could accelerate the adoption of preregistration in in vitro research. Funders have an interest in ensuring that the outcome of their investments is impactful for society and should encourage better research practices by requiring preregistration or providing additional funding to those scientists who undertake this effort. Preregistration of in vitro studies is also consistent with the FAIR principles—making research findable, accessible, interoperable, and reusable - by ensuring that study designs, methods, and data are clearly documented and publicly available. Adherence to these principles meets the expectations of various funding agencies, including the European Commission.

Journals usually come into play only after a study has been completed. Still, aside from offering Registered Reports as an option, by asking for preregistration in reporting checklists, clearly marking preregistered articles as such, or giving them preferential treatment such as prioritized peer-review, journals could contribute to raising awareness among researchers. In turn, preregistered articles could also be an effective way to prevent the publication of fraudulent studies, which often originate from paper mills. These commercial entities that fabricate scientific manuscripts for profit (Sabel et al, 2026) have evolved into highly organized, industrial-scale operations that use artificial intelligence, data fabrication, and manipulate peer-review to infiltrate legitimate journals. The prior deposit of study hypotheses, experimental designs, and analytical workflows in time-stamped, immutable repositories could represent an effective defense mechanism.

Preregistration could also be recognized for evaluating scientists when applying for positions or funding. The Coalition for Advancing Research Assessment (https://www.coara.org/), representing research institutions, research organizations, and funders, seeks measures other than impact factors and number of publications. Preregistration, as a countable unit, may be an indicator of commitment to better research practices.

One structural obstacle to improving the reliability of research is the limited opportunity to publish studies that confirm or refute existing findings. Such studies may struggle to get published in high-impact journals seeking novelty. One possible strategy would be to formally recognize preregistered confirmation or refutation studies that are deposited as preprints. Time-stamped preregistrations linked to preprints could provide verifiable evidence of methodological rigor and research integrity, even if traditional journal publication is delayed or not possible. Funders and institutions could acknowledge preregistered preprints as legitimate scholarly outputs, especially when they confirm or challenge influential findings transparently. This would align incentives with scientific robustness rather than novelty alone.

All these incentives should not be the sole driver of adoption, and emphasis should rather be placed on convincing researchers of the usefulness of preregistration as a means to improve the quality of their work. In the field of psychology, it has achieved significant success without any enforcement, driven by a community effort to improve the reliability of research (https://datacolada.org/115). Therefore, an effort should be made to disseminate the scientific arguments in favor of preregistration for the in vitro research community at an early stage.

The findability of preregistrations is crucial. If researchers must go through several platforms or are not able to search for specific study characteristics, they will be less inclined to use preregistrations to plan their own studies. Therefore, even if the existence of different databases seems worthwhile to meet all needs and to avoid monopolies for this sensitive data, a common searchable platform would be preferable.

The quality check and comparison of the preregistration with the final study report should ideally take place during the peer-review process after submission to a journal. Journals are concerned that this would add further tasks to an already overburdened peer-review system. Structured checklists to help assess internal consistency between hypotheses, design, and analysis plans, or automated tools, could help editors precheck preregistrations and minimize this work for reviewers. In the future, AI tools, such as RegCheck, could help reviewers to identify discrepancies between the protocol and the submitted manuscript (https://regcheck.app/). Alternatively, journals could adopt a lighter-touch approach, such as requiring the disclosure and linking of preregistrations without mandating a full review of the protocol. This would preserve the benefits of transparency without increasing reviewer workload.

In addition, the development of tools following up on preregistrations could help to better address the publication bias. Initiatives monitoring the fate of studies already exist in clinical research (https://eu.trialstracker.net/, https://quest-cttd.charite.de/). Quality indicators for study design and level of detail in reporting could be good measures of improvement (Menon et al, 2026). Demonstrating a measurable improvement in the quality of published results from preregistered trials compared to non-preregistered trials may help convince researchers, funders, and journals of the value of preregistration in general.

## Conclusion and roadmap

Opening up preregistration for in vitro research is a logical step towards improving the quality and transparency of biomedical research. Confirmatory research aimed at informing medical, regulatory, or safety-related decisions may particularly benefit from preregistration, as predefined hypotheses and analysis plans can facilitate critical evaluation and minimize risks for humans. Exploratory fundamental research can likewise benefit from pragmatic preregistration approaches that document key decisions and planned analyses while preserving flexibility.

“Opening up preregistration for in vitro research is a logical step towards improving the quality and transparency of biomedical research.”

To accelerate the adoption of preregistration, we suggest bringing together a task force of researchers from different areas of in vitro research, public registry representatives, funders, journals and regulators. This collaborative strategy could be seen as a lesson learned from animal research, where a missed opportunity for an open-dialog approach probably contributed to the limited uptake of preregistration tools.

As the involvement of many experts could lead to long, laborious, and time-consuming templates, a Delphi approach could help identify essential elements to streamline a template. To further minimize the administrative burden, shorter templates for exploratory studies can be developed for both in vitro and in vivo research. It is crucial to communicate that preregistration aims to promote transparency, not constrain the exploratory nature of science.

**Figure 4:**
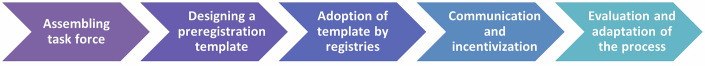
Suggested roadmap for the introduction of specific in vitro preregistration formats. The task force consisting of scientists as well as representatives of journals, funders, and registration platforms work out a preregistration template. In this iterative process, the task force will work on the balance between essential information to increase quality and reproducibility and minimizing the workload. This template should be openly available and could be endorsed by different registries. After implementation, the focus should be on communication and incentivization. After broader uptake, the preregistration process should be evaluated and adapted according to feedback from researchers and stakeholders of the research system.

Finally, making a universal template freely available can encourage different registries to adopt this preregistration form and thereby increase its uptake. Researchers involved in template development can champion preregistration in in vitro research to promote its acceptance and implementation. The process could be initiated by registries or broader networks that advocate for better research practices in biomedical research (Fig. 4).

Implementing in vitro preregistration is a timely and feasible step to strengthen the robustness of novel in vitro methods and accelerate their responsible uptake in basic and translational research.

## Supplementary information

Peer Review File
