# Supplementary material for: A call for preregistration of in vitro research: Pregistration as a tool to improve reproducibility and transparency
Source: EMBO Rep. 2026 Apr 17;27(9):2147–55. doi: 10.1038/s44319-026-00764-x (PMC13171870; doi:10.1038/s44319-026-00764-x)
Supplement: Supplementary file 1 — Peer Review File [file 44319_2026_764_MOESM1_ESM.pdf]

EMBO reports

A call for preregistration of in vitro research: a tool to improve reproducibility and transparency

Céline Heinl, Nuno Franco, Bettina Bert, Katherina Siewert, Dania Movia, Edlira Aruçi, Carina Ladeira, Ahmed Sanin, Winfried Neuhaus, Ulf Kahlert, Manosij Ghosh, Aurélie Thomas, and Athanassia Sotiropoulos

Corresponding author(s): Céline Heinl (celine.heinl@bfr.bund.de)

Review Timeline:	Submission Date:	25th Feb 26
	Accepted:	18th Mar 26

Editor: Holger Breithaupt

Transaction Report: This article was editorially reviewed.
